# Ten simple rules on how to develop a stakeholder engagement plan

**DOI:** 10.1371/journal.pcbi.1010520

**Published:** 2022-10-13

**Authors:** Susanne Hollmann, Babette Regierer, Jaele Bechis, Lesley Tobin, Domenica D’Elia

**Affiliations:** 1 SB Science Management UG (haftungsbeschränkt), Berlin, Germany; 2 University of Potsdam, Faculty of Science, Potsdam, Germany; 3 Leibniz Institute of Vegetable and Ornamental Crops (IGZ) e.V., Großbeeren, Germany; 4 Université de Lorraine, Université de Strasbourg, CNRS, BETA, Nancy, France; 5 Rete Europea dell’Innovazione—REDINN, Pomezia, Italy; 6 Institute for Biomedical Technologies, National Research Council, Bari, Italy

## Abstract

To make research responsible and research outcomes meaningful, it is necessary to communicate our research and to involve as many relevant stakeholders as possible, especially in application-oriented—including information and communications technology (ICT)—research. Nowadays, stakeholder engagement is of fundamental importance to project success and achieving the expected impact and is often mandatory in a third-party funding context. Ultimately, research and development can only be successful if people react positively to the results and benefits generated by a project. For the wider acceptance of research outcomes, it is therefore essential that the public is made aware of and has an opportunity to discuss the results of research undertaken through two-way communication (interpersonal communication) with researchers. Responsible Research and Innovation (RRI), an approach that anticipates and assesses potential implications and societal expectations regarding research and innovation, aims to foster inclusive and sustainable research and innovation. Research and innovation processes need to become more responsive and adaptive to these grand challenges. This implies, among other things, the introduction of broader foresight and impact assessments for new technologies beyond their anticipated market benefits and risks. Therefore, this article provides a structured workflow that explains “*how to develop a stakeholder engagement plan*” step by step.

## Introduction

Scientific knowledge is one of the pillars of the modern economy [1]. As underlined by the European Commission (EC) [2], science strongly contributes to innovation creation and valorisation [3]. Innovation, as the dissemination, reuse, and valorisation of knowledge, plays a major role in employment and economic growth. While the time lag between research and its financial economic exploitation may be long and characterised by high uncertainty, “the economic impact of science is indisputable” [4].

For these reasons, funding bodies playing a major role in research funding, such as the EC, have always been interested in the optimal spending on scientific research [5] and continue to orient policies for making research outcomes more meaningful, and research more responsible. This applies to basic scientific research as well as to applied science. Citing Needham, Freeman says that “There is really only science with long term promise of application and science with short term promise of application” [6].

Meaningful and responsible research requires the alignment of public-funded research with societal values and needs to influence the project’s trajectory and increase the societal impact. Research and technological advances only make sense if they are useful, usable, and embraced by consumers. This requires the public to be made aware of new scientific developments and to have the opportunity to communicate and interact with researchers. Therefore, early engagement with stakeholders is of paramount importance [7–9].

Responsible Research and Innovation (RRI) emphasises the importance of two-way communication between researchers and stakeholders. It is a “transparent, interactive process by which societal actors and innovators become mutually responsive to each other with a view on the (ethical) acceptability, sustainability and societal desirability of the innovation process and its marketable products” [7]. “RRI should be understood as a strategy of stakeholders to become mutually responsive to each other and anticipate research and innovation outcomes underpinning the ‘grand challenges’ of our time for which they share responsibility” [10] and as such lead to desirable societal benefits [11,12].

**Fig 1:**
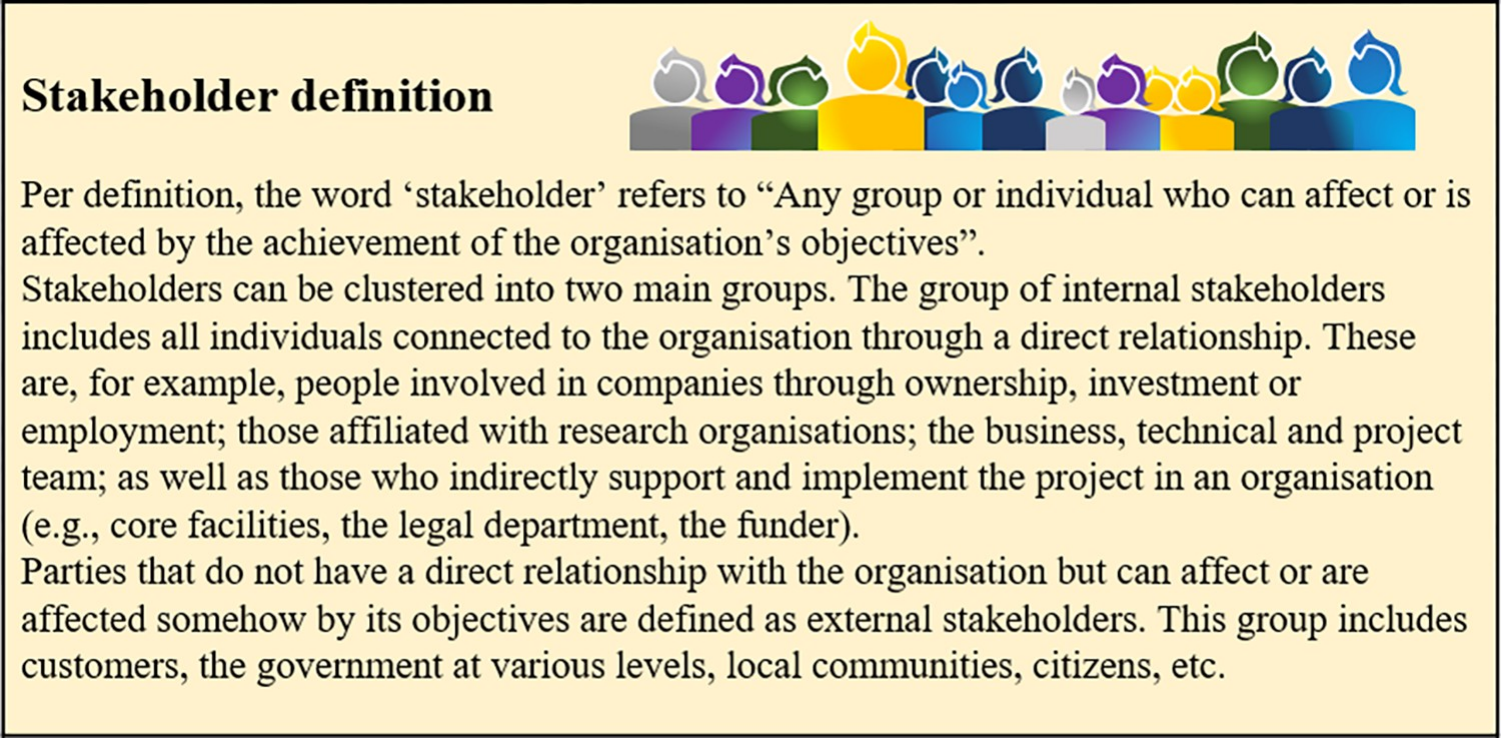
What is a stakeholder and who belongs to it? Definition of a stakeholder [6].

In this context, engagement with multiple stakeholders (Fig 1) is of utmost importance in modern research for catalysing scientific knowledge use [13,14] especially through information and communications technology (ICT).

In addition, allowing stakeholders to interact with the project in its initial stage can result in tailored products, services and solutions [15,16]. This engagement allows the researchers to adjust project strategies from the beginning, avoiding futile, money-wasting, and time-consuming activities. Therefore, since 2014, the EC has called for engagement with stakeholders as a prerequisite to receiving funding.

To discuss the research results, two-way communication requires engaging stakeholders, which may not be an easy task. The added value, however, is that it allows both the communicator and the recipient to freely express their views, ideas, and feelings. This mutual exchange of information creates a democratic environment in the setting that is beneficial for both parties. For the project team, it is an opportunity to receive relatable feedback and, if needed, to react accordingly. For stakeholders, it represents a way to participate in the whole research project and, in this way, guide the research towards its specific objectives and real needs.

Due to the heterogeneity of stakeholders, the implementation of different approaches is necessary to reach every single actor and ensure appropriate coordination.

With this paper, we provide “10 simple rules on how to develop a stakeholder engagement plan” that help identify and manage stakeholder engagement and, fundamentally, adopt an RRI approach. It is important to note here that the stakeholder engagement plan is not a static document and should be revised and reviewed in tandem with the project implementation.

## Results

### Rule 1: Identify and formulate the challenges of the project

Before identifying the stakeholders, a careful analysis of the internal and external opportunities and threats or risks to the project should be carried out. This analysis of the project framework conditions is necessary to achieve its expected outcomes and impact and to understand which stakeholder groups should be involved for the success of the project. In doing so, 2 main tools can help to analyse both internal and external challenges to be overcome in order to reach the desired outcomes. The Strengths, Weaknesses, Opportunities, and Threats (SWOT) Analysis tool [17] is designed for the identification of both internal and external factors that may influence project outcomes and impact. In particular, the tool focuses on strengths and weaknesses that characterise the project’s success and on opportunities and threats that emanate from the external environment. Because of the complexity of the external environment, the analysis needs to be further developed using the Political, Economic, Social, Technological, Environmental, and Legal (PESTEL) [17] framework. This second tool focuses on the external elements, namely political, economic, social, technological, environmental, and legal factors that may influence the desired outcomes. The identification of these key external factors is crucial because they might have a relevant influence on your research, while you have only little or no means to control these. With a careful analysis of these factors, it is possible to take action to avoid potential pitfalls and, on the other hand, exploit new opportunities.

By conducting the analysis, elements are identified that provide a clearer view of such factors and conditions that may affect the project outcomes. Knowing them helps to identify and select the conditions that need to be met in order to achieve your objectives, including the stakeholders to be involved.

### Rule 2: Identify stakeholders

Once the SWOT and PESTEL analysis results are specified, it is easier to identify actors, i.e., mapping stakeholders, that are likely to be impacted by project outcomes. In this second step, it is necessary to select the actors that need to be involved in facing the defined challenges. To avoid investing time and efforts in a nontargeted (or unfavourable) strategy, it is crucial to ensure that relevant stakeholders are already engaged from the beginning of the research activities.

It is advisable to commence with the specification of the stakeholder reference groups (the stakeholder areas). Stakeholder areas describe the fields that stakeholders represent, i.e., Research, Industry, Policy, and Society. We further divided these stakeholders’ areas into 3 more specific levels: macro-, meso-, and microlevel. The authors of this paper define these levels as follows: macrolevel means all individuals directly affiliated or working in a branch of a stakeholder area such as, e.g., the pharmaceutical industry without any further specification or discrimination. They can be technicians, project managers, engineers, or persons from the management area. Although they belong to the same area, they will not have the same background, needs, interests, or influence on the project. Mesolevel refers to groups representing specific sectors of the macrolevel: one sector covers, for example, all technicians and manufacturing people, the developers, the managers, etc., representing another sector. The microlevel is represented by the single individuals from the above mesolevel.

Keeping in mind the immediate needs of the project, the next stage entails scheming out potential stakeholder needs and the expected impact on the project outcomes as a result of their involvement (feedback).

This analysis enables the evaluation and comparison of the different effects of specific stakeholders on the project as well as how the project can meet diverse stakeholder needs or expectations. The data should be structured in a table designed to record 3 types of key data for each stakeholder; for example, stakeholder needs, project needs, and expected impact.

### Rule 3: Implement a roadmap to ensure conformity with data privacy policy

Intellectual property (IP) concerns and data disclosure are examples of issues to be addressed when interacting with actors involved in the project in different ways. While IP refers to the outgoing data management, data disclosure concerns incoming data. Confidentiality of project data is a typical example of data that may raise IP concerns when they are to be shared with stakeholders. In contrast, the management of data generated by stakeholders clearly represents a data disclosure issue.

Therefore, an essential element here is the implementation of specific measures, ensuring conformity with the existing regulations at both national, supranational, and international levels. An example is the General Data Protection Regulation (GDPR) [18], applicable to all entities collecting data related to persons in the European Union. GDPR is developed around 4 main axes: lawful basis and transparency, data security, accountability and governance, and privacy rights. For example, the data minimisation principle, included in the data security section, states that “You should collect and process only as much data as necessary for the purposes specified.” Because interaction measures differ between stakeholder groups, the information provided to or needed from each stakeholder group might be different. It may be that, for example, information about the age and ethnicity of stakeholders is important for one group but might not be essential for another. Therefore, in order to comply with the data minimisation principle, it is vital to form a clear idea about the specific data emanating from each stakeholder group that is required for the project.

Following the identification of all the IP and data management aspects to be addressed, the next step is to define a robust strategy for complying with the legal framework, e.g., data transfer agreements, consent agreements, etc. As a legal matter and one of the pillars of RRI, from this point, a Data Protection Officer, or the Technology Transfer Office, who also manage ethical issues, must be involved in this process.

### Rule 4: Collect stakeholder data (stakeholder register)

All stakeholder information should be collected in a stakeholder register (Table 1). The personal information entered in the Register forms the basis for the stakeholder mapping, whereby all individuals are assigned to a specific area and level. It is important to note that some individuals can belong to different stakeholder groups.

**Table 1:** Example stakeholder register.

First name	Surname	Email	Address	Profession	Roles and responsibilities	Area	Relation with the project (internal/external)
							

The stakeholder register should record the following

• The stakeholder’s name, surname, and contact data

• The stakeholder’s relationship with the project, which is either internal or external

• The stakeholder’s profession

• The stakeholder’s role/position and responsibilities

### Rule 5: Categorise stakeholders into priority groups

Once the stakeholder register is compiled, stakeholders can be categorised into different priority groups.

Stakeholder classification is necessary for managing their engagement. Stakeholder engagement is a time-consuming activity, and stakeholders differ in terms of the role and the influence they may have on the project’s success. For example, high-priority group members can support or hinder the project, while low-priority groups have a marginal impact on the project outcomes. Therefore, it is essential to prioritise some groups over others.

An effective tool for prioritising stakeholder groups is Mendelow’s matrix [19]. This system analyses stakeholders according to 2 main characteristics: their interest in the project and their influence on the project outcomes. As a result, 4 different priority groups will emerge: (1) Key stakeholder; (2) Influencer; (3) Interested stakeholder; and (4) Passive stakeholder. The key stakeholders are individuals with high interest and high influence. Influencers are those who have low interest but high influence. The 2 remaining groups are the interested (high interest/low influence) and passive stakeholders (low interest/low influence). Results of stakeholder categorisation are provided in Table 2.

**Table 2:** Stakeholder categorisation.

First name	Surname	Email	Address	Profession	Roles and responsibilities	Area	Relation with the project (internal/external)	Stakeholder category
								

Once the stakeholders have been categorised into priority groups, the matrix enables the definition of the purpose and the degree of the interaction with each stakeholder group. Special attention must be paid to those individuals who belong to the first 2 groups.

When the purpose is clarified for each stakeholder, stakeholder engagement efforts and activities can be oriented, as shown in Table 3, where the categorisation output is reported in the first column while the engagement efforts and activities are displayed in the third column.

**Table 3:** Key actions to engage with the different stakeholder categories.

Category	Influence/Interest	Key actions towards such stakeholders
Key stakeholders	High influence and high interest	Objective: To collaborate and manage this group. Engage at the earliest possibility. Continuous communication built by sending project updates, consulting their opinions, inviting them to events, etc.
Influencers	High influence but low interest	Objective: To keep this group’s needs satisfied. Efforts need to be made to ensure that they are key stakeholders. Communication actions stressing the project’s benefits and raising curiosity.
Interested stakeholders	Low influence but high interest	Objective: To keep this group informed. Continuous communication to inform them about project progress, actions, and results. Potential consultation regarding areas of stakeholder interest (especially regarding specific questions or uncertainties that the project faces).
Passive stakeholders	Low influence and low interest	Objective: To monitor this group with minimum effort. No specific actions need to be taken to address this group. Might be informed through general communication actions of the project (e.g., website, newsletter).

It is vital to remember that priority groups may change over time, depending on the project’s needs and progress. If this occurs, the process should be reiterated so that required adjustments can be made.

### Rule 6: Devise a stakeholder engagement plan

After creating the stakeholder register, followed by the mapping and prioritisation of the stakeholder groups, the next stage is to devise your stakeholder engagement plan. This plan is designed as a strategic document and timeline that will facilitate the management of stakeholder engagement throughout the lifespan of the project.

As a strategic document, the plan outlines all the specific activities addressed to each stakeholder group that fit the purposes identified in Rule 5. Because the nature of stakeholder involvement is not the same for each priority group, different strategies and media tools must be adopted to reach and engage them. For example, for key stakeholders, solely issuing and targeting them with newsletters will be insufficient and other strategies should be added and implemented to keep them on track, such as round tables and workshops. Conversely, for passive stakeholders, social networks and newsletters are adequate. The greater the stakeholder’s interest and power, the greater the communication flow should be.

Identifying the instruments to be used for implementing the stakeholder engagement plan or strategy is a key point concerning the methodology to be developed. To this end, it is worthwhile listing all the possible dissemination tools considered most suitable for the project’s communication strategy, and then deciding which group(s) can be effectively reached with each tool. Moreover, communication should be two-way; therefore, stakeholders must also be able to communicate with the project representatives and members.

Once the communication strategy has been finalised, it is time to focus on planning activities. Given the different activities necessary for engaging stakeholders, this plan should also be designed along a timeline. Scheduling activities on a timeline will facilitate their timely implementation and allow for appropriate priority to be given to each stakeholder group to be contacted and engaged, in line with the project schedule and deliverable deadlines.

All the activities necessary for implementing the stakeholder engagement plan require time and human as well as other resources. Knowing how much time is required to complete a task or a series of tasks (e.g., preparing a survey or organising a meeting) is pivotal for the plan to be successful. Therefore, in compiling and fixing the schedule of planned activities, the human resources that must be dedicated and the time necessary for each activity should be carefully calibrated. For example, preparing a round table or a workshop requires far more time, resources, and effort than preparing a survey or creating a brochure. For the organisation of a meeting, there are several key considerations to be made. These include topic selection (depending on the high-priority group interests), identification and invitation of keynote speakers, preparation of publicity material (e.g., brochure or flyer), dissemination of the event on selected channels (newspapers, social media, etc.), selection of the event venue, hosting, and facilitation, and in some cases the organisation of social events that are fundamental to prompt interpersonal relationships and stimulate people to work together towards common objectives. Such activities require planning months in advance, with each planning stage executed on time. Finally, since stakeholders are often not members of the project, they may need to be motivated to foster active participation in the organised activities. They may not always be available to participate throughout the planned schedule. Moreover, stakeholders also have individual preferences; for example, some prefer meetings while others like to engage in a one-on-one conversation or through written media; also, security or IP issues might play a role when choosing the optimal interaction tool. Thus, the engagement plan should be adaptable and flexible with built-in alternative strategies if stakeholders prove to be unresponsive.

### Rule 7: Identify, select, and test tools for the implementation of Rule 6 and its GDPR conformity

Once the communication strategy has been defined and the most suitable tools to be used identified, it is time to work on the content and format of the chosen communication media. This will require identifying the communication tool that best fits the project’s needs and is the most appealing to the target stakeholder group. During this phase, it is important to define the approach for communication, the content, and the tool(s) to be used. For example, choices may include face-to-face or online meetings, newsletters, surveys, structured interviews, and social network campaigns. Of these choices, structured interviews can prove beneficial in that the questions are planned and created in advance. Accordingly, all candidates are asked the same questions in the same order, making it easier to compare and evaluate their responses objectively and fairly, rendering such structured interviews more legally defensible.

Once the communication media have been compiled, it is worth conducting a meaning and error check for clarity and correctness. A review by peers and colleagues may improve the comprehensibility and accuracy of the media, its formulation, design, and hence the overall efficacy of the communication strategy.

The final step involves testing the GDPR conformity of the communication media, referring to the strategy defined when following Rule 3. The collected interview data and the tools and software utilised for managing the communication activity should be examined for compliance with GDPR guidelines.

### Rule 8: Make contact—Engage stakeholders

**Fig 2:**
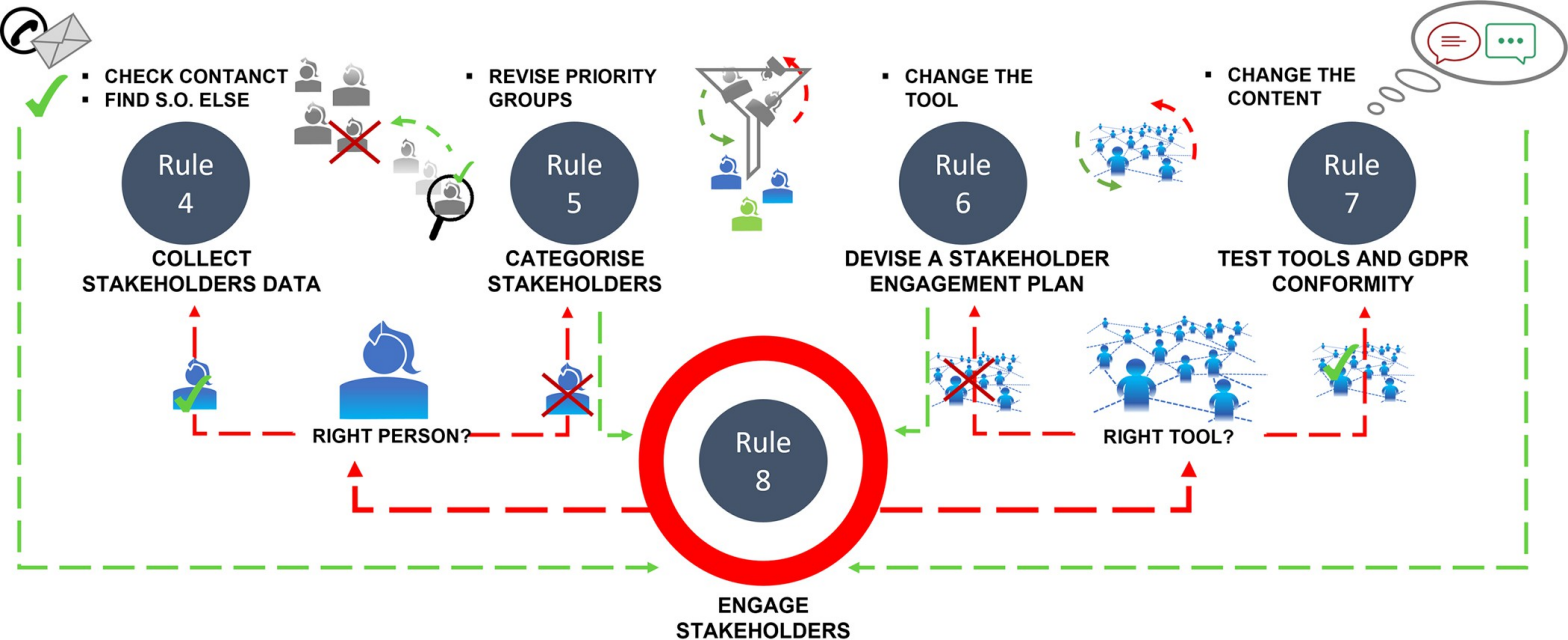
How to revise the stakeholder engagement strategy. If the stakeholder engagement is not successful, it may be necessary to revisit previous rules and, especially, to check contacts, replace unresponsive stakeholders, and change the content and/or the communication flow. The scheme demonstrates the workflow for revision.

At this point, the engagement plan can be rolled out to initiate the two-way communication with the stakeholder groups, and this communication should be followed through. For example, when an email is sent to a stakeholder, it is important to pursue this line of contact and ascertain whether a connection has indeed been established, i.e., whether the targeted stakeholder responded positively to the email, subscribed to the project newsletter, or, conversely, if they unsubscribed from your mailing list. Different factors may affect the success of the engagement: For example, unresponsiveness may be an indication that the group has been wrongly prioritised or the person to engage with has been misidentified, or an inadequate media channel has been selected and utilised. Ultimately, it may be that the email sent has landed in their junk folder or was filtered by a spamming programme or firewall. Should these issues arise, it may be necessary to revisit the previous rules (Fig 2) and make the necessary adjustments until a satisfactory result is achieved. The number of responses or newsletter subscriptions will be strong indicators as to whether a trusting relationship has been built with the stakeholders.

Note that it is not yet a central task to include all external stakeholders in the communication flow at the beginning of a project. However, the extensive exploitation of social networks is central to the initiation of the communication process. One starting point is to create posts on media channels where specific interest groups or communities can be targeted, for example, scientific networking tools such as ResearchGate, or professional networking platforms such as LinkedIn. Posting project-related videos on YouTube can also increase visibility, particularly if the video is well designed and the content is developed by experts in the field. In this type of promotion for the project, the appeal of the communication can be enhanced with the use of nonspecialist language and visually attractive material.

### Rule 9: Evaluate stakeholder feedback

Considering stakeholder responses is not a passive task, but an assessment activity. Stakeholder feedback should be discussed with partners and, if appropriate, with other stakeholders before it is incorporated into the project strategy leading, e.g., to changes in the project plan. The purpose of stakeholder involvement is to make the research successful and responsible and to balance the needs and requirements of the project with those of the stakeholders.

Once stakeholders are involved, the extent and nature of their response can be assessed. Imagine the scenario where a new app is being developed. In this case, it is reasonable to include potential users in the list of stakeholders. You then apply Rule 8 and invite them to participate in a survey. The survey shows that your app is well received in principle, but users do not feel comfortable using it, which makes it unattractive. Before changing the project plan, it might be advisable to formulate acceptance criteria and consult the stakeholders, as this increases the likelihood that the customers’ expectations—and not the developers’ needs—are met. If necessary, additional requirements can be identified and discussed with stakeholders or a developer before implementation.

If opinions are divergent, reiteration of the evaluation process clarifies positions and perspectives to determine if the requested change should be implemented in the project strategy or not. This evaluation process will also further enhance the two-way communication process. Another way to evaluate stakeholders’ reaction is by using data from some of the tools identified in the communication strategy (Rule 7). Different software exists that allows to retrieve information from stakeholders’ behaviour, such as when they receive the newsletter, respond to questionnaires, or visit the website. GDPR-compliant newsletter tools such as rapidmail, klicktipp, or GetResponse offer an anonymised automatic evaluation of recipient behaviour. This includes information on opens, bounces, clicks on provided links, and unsubscribes. Such software also helps ascertain user tools and demographics, such as whether the message was opened from a desktop, tablet, or phone and in which country, and the gender balance. When performing face-to-face events such as workshops or meetings, afterwards, the administration of a survey on the level of satisfaction or the level of commitment of attendants is of utmost importance to test the effectiveness of the activity and the success of the event for the project objectives. This monitoring is central to the continuous improvement of the communication strategy until it is possible to achieve the desired stakeholder group represented in sufficient numbers and statistically relevant.

### Rule 10: Adjust engagement plan

**Fig 3:**
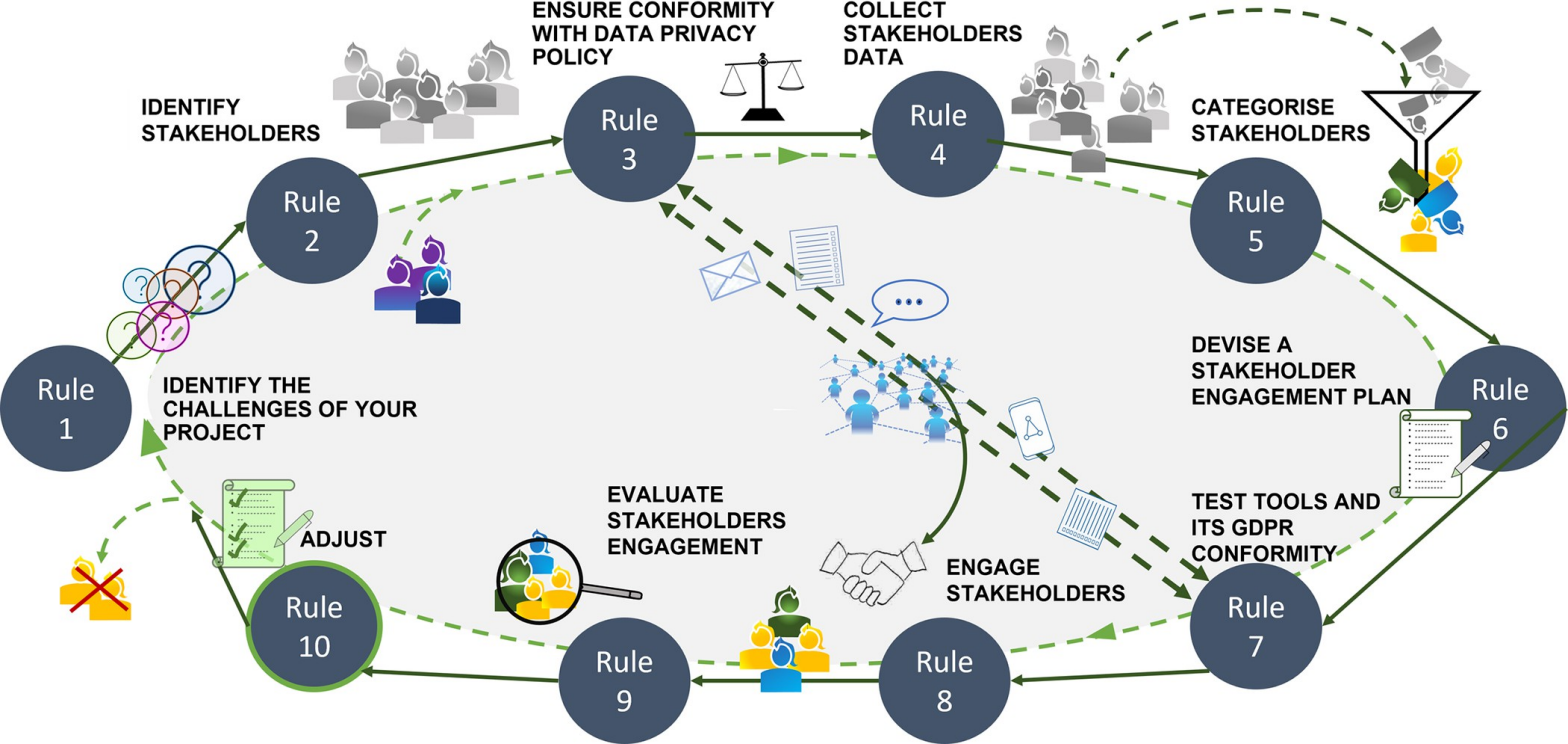
Ten simple rules to develop a stakeholder engagement plan. Workflow. The scheme demonstrates an optimal workflow for the generation of a stakeholder engagement plan including the preparatory, implementation, and follow-up phase for a complete cycle.

After evaluating stakeholder feedback, it is finally time to adjust the project to best fit its objectives, thereby making research outcomes more meaningful and research more responsible. Once the project plans have been revised, the entire process should be assessed from the start and, if necessary, adjusted where tensions were found (Fig 3). This can either be the case at rule 1 (as shown in Fig 2), e.g., if the framework conditions of the project have changed, or at any later stage in the process. The purpose of this process is to continuously improve processes and accelerate the use of a system under development to minimise risks and undesirable developments similar to the OODA loop (Observe-Orient-Decide-Act cycle) [20], PDCA (plan-do-check-act or plan-do-check-adjust) [21], and agile software development [22]. The significance of this should not be underestimated: Early and timely project changes can prevent the loss of time and resources.

It is also critical to the project’s success that results are continually measured and evaluated if they are still aligned with stakeholder needs based on the project objectives. As mentioned, corrective actions should be implemented as early as possible as this will prevent small issues from becoming larger and irreparable problems. Table 4 provides a nonexhaustive list of questions to help in designing the stakeholder engagement plan.

**Table 4:** Nonexhaustive list of questions for developing a stakeholder engagement plan.

Rule	Aim	Questions
**Rule 1**	Identify and formulate the challenges of the project	• What are the desired outcomes of the project? • What are the internal strengths/weaknesses? • What are the external opportunities/threats? • What are the environmental factors that should be taken into account?
**Rule 2**	Identify stakeholders	• Who are the stakeholders in the different areas? • Who are the stakeholders at macro-, meso-, and microlevel? • What are the project needs? • What are the stakeholder needs? • What is the expected impact of involving the stakeholder?
**Rule 3**	Implement a roadmap to ensure conformity with data privacy policy	• What data are required? • To which data will the stakeholder have access? • To which data will other partners have access? • What software will be deployed? • Are there any IP and data disclosure concerns?
**Rule 4**	Collect stakeholder data (stakeholder register)	• Who is the stakeholder? • Which is the stakeholder relationship? • What is the stakeholder role? • Who is the stakeholder contact? • To which areas/levels does the stakeholder belong?
**Rule 5**	Categorise stakeholders into priority groups	• Is the stakeholder interested in the project? • Does the stakeholder have a strong influence on the project outcomes? • To which group does the stakeholder belong? • What is the objective of the future communication/interaction?
**Rule 6**	Devise a stakeholder engagement plan	• What are the tools for reaching the stakeholders? • Which tools are useful for reaching a particular stakeholder? • When should stakeholders be engaged? • What is the frequency of the engagement?
**Rule 7**	Identify, select, and test tools for the implementation of Rule 6 and its GDPR conformity	• How should the tool be designed? • Do the tools work well? • What do testers think about the tools? • How can the design be improved?
**Rule 8**	Make contact—Engage stakeholders	• Have stakeholders been reached? • Has the right person been reached? • Does the prioritisation of stakeholders need to be modified? • Does the communication strategy need to be modified?
**Rule 9**	Evaluate stakeholder feedback	• Has feedback been transferred to partners? • How have reactions been evaluated? • Do stakeholder reactions need to be deeply analysed? • Have stakeholders been informed about recent changes, according to their needs?
**Rule 10**	Adjust engagement plan (restart process from Rule 1)	• Does the project need to be revised? • Are the project needs and stakeholder needs aligned with each other now?

## Conclusions

Nowadays, stakeholder participation in research projects has become a more and more common practice. Usually required by third-party–funded projects, their engagement is of utmost importance to render the research more responsible and research outcomes more meaningful. For example, in solution-oriented research, such as in systems medicine, the effective involvement of stakeholders in the research process is fundamental. Here, one prominent example is personalised medicine, for which the patient is the core reference. What the patients eat, their lifestyle, the type and degree of stress they experience, and the environmental elements to which they are exposed constitute core data to be collected, analysed, and meaningfully correlated to clinical indicators. This is the reason why the involvement of stakeholders is so important. However, dealing with stakeholders is a complex process. Which stakeholders need to be involved in the project? How can they be reached? How can they be engaged? Are they equally relevant for the expected outcomes of the project?

Having an effective stakeholder engagement plan is necessary for successfully identifying, mapping, prioritising, and engaging focal actors who will have the power to positively influence your project trajectory and outcomes. These 10 simple rules will facilitate the development and implementation of a stakeholder engagement plan, monitor the evolution of the importance of the stakeholder groups, and make any necessary adjustments during the lifespan of a project. A robust and effective engagement plan will optimise and maximise the impact of your project.

## References

[1] Foray D. L’économie de la connaissance. La Découverte. Repères. 2000. Available from: https://www.cairn.info/l-economie-de-la-connaissance—9782707197573.htm.

[2] Hilty R, Krujatz S, Bajon B, Frueh A, Kur A, Drexl J, et al. European Commission—Green Paper: Copyright in the Knowledge Economy—Comments by the Max Planck Institute for Intellectual Property, Competition and Tax Law [Internet]. Rochester, NY: Social Science Research Network; 2008 Dec. Report No.: ID 1317730. Available from: https://papers.ssrn.com/abstract=1317730.

[3] Coccia M. Evolution of the economics of science in the Twenty Century. J Econ Libr. 2018 Mar 18;5(1):65–84.

[4] Stephan PE. The Economics of Science. J Econ Lit. 1996;34 (3):1199–1235.

[5] Nelson R. The Simple Economics of Basic Scientific Research. J Polit Econ. 1959;67. Available from: https://econpapers.repec.org/article/ucpjpolec/v_3a67_3ay_3a1959_3ap_3a297.htm.

[6] Freeman RE. Strategic management: A stakeholder approach. Boston, Pitman. 1984

[7] Von Schomberg R. Towards Responsible Research and Innovation in the Information and Communication Technologies and Security Technologies Fields (November 13, 2011). Available from: https://ssrn.com/abstract=2436399.

[8] Carter SK, Pilliod DS, Haby T, Prentice KL, Aldridge CL, Anderson PJ, et al. Bridging the research-management gap: landscape science in practice on public lands in the western United States. Landsc Ecol. 2020;35 (3):545–560.

[9] Naugle DE, Allred BW, Jones MO, Twidwell D, Maestas JD. Coproducing science to inform working lands: The next frontier in nature conservation. BioScience. 2020;70 (1):90–96. doi: 10.1093/biosci/biz144 31949318 PMC6956880

[10] Von Schomberg R. A vision of responsible research and innovation. Responsible Innovation: Managing the Responsible Emergence of Science and Innovation in Society. John Wiley & Sons: Hoboken, NJ, USA; 2013. p. 51–74.

[11] Owen R, Macnaghten P, Stilgoe J. Responsible research and innovation: From science in society to science for society, with society. Sci Public Policy. 2012;39(6):751–760.

[12] Genus A, Stirling A. Collingridge and the dilemma of control: Towards responsible and accountable innovation. Res Policy. 2018;47(1):61–69.

[13] Freedman P. The Principles of Scientific Research. London: Macdonald & Co. Ltd; 1949.

[14] Arnott JC, Mach KJ, Wong-Parodi G. Editorial overview: The science of actionable knowledge. Curr Opin Environ Sustain. 2020 Feb 1;42: A1–5.

[15] Bamzai-Dodson A, Cravens AE, Wade AA, McPherson RA. Engaging with stakeholders to produce actionable science: a framework and guidance. Weather Clim Soc. 2021;13(4):1027–1041.

[16] Stahl BC, Obach M, Yaghmaei E, Ikonen V, Chatfield K, Brem A. The responsible research and innovation (RRI) maturity model: Linking theory and practice. Sustainability. 2017;9(6):1036.

[17] SWOT and PESTEL Analysis: An Overview by Jaele Bechis [Internet]. 10.5281/zenodo.6603058.

[18] GDPR [Internet]. What is GDPR, the EU’s new data protection law? [cited 2022 Mar 13]. Available from: https://gdpr.eu/what-is-gdpr/.

[19] Mendelow A. Stakeholder Mapping. Proceedings of the 2nd International Conference on Information Systems. Plenum Publishers: Cambridge, MA; 1991.

[20] Brehmer B. The Dynamic OODA Loop: Amalgamating Boyd’s OODA Loop and the Cybernetic Approach to Command and Control ASSESSMENT, TOOLS AND METRICS. 2005. Available from: https://www.semanticscholar.org/paper/The-Dynamic-OODA-Loop-:-Amalgamating-Boyd-’-s-OODA-Brehmer/7e9d23a6911d636666338358505613bb5eba43b8.

[21] Lewis WE. PDCA/Test: A Quality Framework for Software Testing. 1st ed. 1998. Auerbach Publications. 10.1201/9780429131769.

[22] Cockburn A. Agile Software Development. 2001. Available from https://www.semanticscholar.org/paper/Agile-Software-Development-Cockburn/2ca53807f49c49f82673273b8b10658c3cb032c6.

[23] cost-charme.eu [Internet]. COST Action Harmonising standardisation strategies to increase efficiency and competitiveness of European life-science research (CHARME); c2016-2020 [cited 2020 Apr 7]. Available from: https://www.cost-charme.eu/.

[24] oxipro.eu [Internet]. Transition towards environment-friendly consumer products by co-creation of an oxidoreductase foundry. Horizon Europe GA Grant agreement ID: 101000607 [cited 2022 May 27]. Available from: https://www.oxipro.eu/.

